# Supplementary material for: Fitness Cost of blaNDM-5-Carrying p3R-IncX3 Plasmids in Wild-Type NDM-Free Enterobacteriaceae
Source: Microorganisms. 2020 Mar 7;8(3):377. doi: 10.3390/microorganisms8030377 (PMC7143814; doi:10.3390/microorganisms8030377)
Supplement: Supplementary file 1 [file microorganisms-08-00377-s001.pdf]

Table 1. Numbers of recipient strains from different sources.

Source	Species							
	E. coli	*K. pneumoniae*	*Providencia*	*Enterobacter cloacae*	*Citrobacter freundii*	*Proteus mirabilis*	*Klebsiella oxytoca*	Total
Environment	3	3	1	1	-	-	-	8
Chicken	3	3	-	-	-	1	-	7
Meat	3	1	-	-	1	-	1	6
Fly	2	1	1	1	-	-	-	5
Human	6	-	-	-	-	-	-	6
Swine	3	3	-	-	-	-	-	6
Companion animal	3	-	-	-	-	-	-	3
Total	23	11	2	2	1	1	1	41

Table 2. Detailed information on recipient strains and conjugative transfer results.

Number	Species	Sources	ST	Conjugative transfer	Number	Species	Sources	ST	Conjugation transfer
1	*E. coli*	chicken manure compost	354	√	22	*E. coli*	fly on pig farm	871	√
2	*E. coli*	chicken manure compost	224	√	23	*E. coli*	fly on chicken farm	744	√
3	*E. coli*	chicken manure compost	43	√	24	*K. pneumoniae*	fly on chicken farm	11	×
4	*K. pneumoniae*	chicken feces	11	√	26	*Enterobacter hormaechei*	fly on chicken farm	7742	√
5	*K. pneumoniae*	soil in chicken farm	789	×	27	*Providencia*	fly on chicken farm	NF*	×
6	*K. pneumoniae*	soil in chicken farm	789	×	28	*E. coli*	human	131	√
7	*Enterobacter cloacae*	chicken manure compost	NF*	√	T1	*K. pneumoniae*	swine	2981	√
8	*Providencia*	chicken manure compost	NF*	×	T2	*K. pneumoniae*	swine	661	×
9	*E. coli*	chicken cloaca swab	2732	×	T3	*K. pneumoniae*	swine	1086	√

10	*E. coli*	chicken cloaca swab	1564	√	A19	*E. coli*	swine	542	√
11	*E. coli*	chicken cloaca swab	156	√	C45	*E. coli*	swine	216	√
12	*Proteus mirabilis*	chicken cloaca swab	NF*	×	D22	*E. coli*	swine	206	√
13	*K. pneumoniae*	chicken cloaca swab	11	√	0970	*E. coli*	companion animal	405	√
14	*K. pneumoniae*	chicken cloaca swab	395	√	1150	*E. coli*	companion animal	10	×
15	*K. pneumoniae*	chicken cloaca swab	11	√	10194	*E. coli*	companion animal	617	√
16	*E. coli*	pork from farmers market	542	√	S43	*E. coli*	human	1415	×
17	*E. coli*	chicken from supermarket	2606	×	S51	*E. coli*	human	359	√
18	*E. coli*	chicken from supermarket	58	√	S69	*E. coli*	human	744	√
19	*Raoultella planticola*	chicken from supermarket	NF*	√	S73	*E. coli*	human	190	×
20	*K. pneumoniae*	pork from retail store	NF*	√	S87	*E. coli*	human	90	√

21	*Citrobacter freundii*	chicken from farmers market	NF*	√	
Total stains: 41		Conjugative transfer successfully: 29			Conjugative transfer rate: 70.7% (29/41)

√ indicates successful conjugative transfer; × indicates that conjugative transfer was unsuccessful; NF*, not found.

Table 3. Conjugative transfer success rates in different species.

Species	Total	Conjugation transfer successfully	Rate
E. coli	23	18	78.3%
K. p.	11	7	63.6%
Others	7	4	57.1%
Total	41	29	70.7%

Table 4. Conjugative transfer success rates among strains from different sources.

Source	Total	Conjugative transfer successfully	Rate
Environment	8	5	62.5%
Chicken	7	5	71.4%
Meat	6	5	83.3%
Fly	5	3	60.0%
Human	6	4	66.6%
Swine	6	5	83.3%
Companion animal	3	2	66.6%
Total	42	29	70.7%

Table 5. Genome and plasmid information for donor strain *Escherichia coli* 3R.

	CDs	Length (KB)	Plasmid typing	Resistance genes	Secretion systems
Genome	4632	4886.9		*tet*(B), *dfrA12*, *floR*, *fosA*, *bla*$_{CTX-M-65}$	Type II
Plasmid1	219	193.0	IncFIB		
Plasmid2	110	94.9	IncI1	*bla*$_{CMY-2}$	

Plasmid3	81	67.6	IncFII	*bla*$_{TEM-1}$		
Plasmid4	61	46.1	IncX3	*bla*$_{NDM-5}$		Type IV

Table 6. Specific primers targeting the four plasmid types in *Escherichia coli* 3R.

Primer name	Sequence(5'-3')
P1-F	TGCACGAGTGGGTTACATCG
P1-R	ACGCTCGTCGTTTGGTATGG
P2-F	TCCGGTCACGAAATACTGGC
P2-R	TTTACCTCAACGGCGGGAAG
P3-F	ACGTACTGATGCCACGGAAG
P3-R	TACAGTTCAAACAGGCGGGG
P4-F	GGTTTGGCGATCTGGTTTTC
P4-R	CGGAATGGCTCATCACGATC

Table 7. The Genbank accession numbers of strains. The complete genome sequences data of all tested strains in this study have been uploaded to NCBI database. In order to identify the strains, "M-" was added to all strain names before their previous names.

Strain name	Accession number	Strain name	Accession number	Strain name	Accession number
M-1	JAALCP000000000	M-28	JAALBS000000000	M-15-X3	JAALAU000000000
M-2	JAALCO000000000	M-T1	JAALEZ000000000	M-16-X3	JAALAT000000000
M-3	JAALCN000000000	M-T2	JAALBR00000000	M-18-X3	JAALAS000000000
M-4	JAALCM000000000	M-T3	JAALBQ000000000	M-19-X3	JAALEY000000000
M-5	JAALCL000000000	M-A19	JAALBP000000000	M-20-X3	JAALEX000000000
M-6	JAALCK000000000	M-C45	JAALBO000000000	M-21-X3	JAALAR000000000
M-7	JAALCJ000000000	M-D22	JAALBN000000000	M-22-X3	JAALAQ000000000
M-8	JAALCI000000000	M-0970	JAALBM000000000	M-23-X3	JAALAP000000000
M-9	JAALCH000000000	M-1150	JAALBL000000000	M-26-X3	JAALAO000000000
M-10	JAALCG000000000	M-10194	JAALBK000000000	M-28-X3	JAALAN000000000
M-11	JAALCF000000000	M-S43	JAALBJ000000000	M-T1-X3	JAALEW000000000
M-12	JAALCE000000000	M-S51	JAALBI000000000	M-T3-X3	JAALAM000000000
M-13	JAALCD000000000	M-S69	JAALBH000000000	M-A19-X3	JAALAL000000000
M-14	JAALCC000000000	M-S73	JAALBG000000000	M-C45-X3	JAALAK000000000
M-15	JAALCB000000000	M-S87	JAALBF000000000	M-D22-X3	JAALAJ000000000
M-16	JAALCA000000000	M-DH5a	JAALBE000000000	M-0970-X3	JAALAI000000000
M-17	JAALBZ000000000	M-1-X3	JAALBD000000000	M-10194-X3	JAALAH000000000
M-18	JAALBY000000000	M-2-X3	JAALBC000000000	M-S51-X3	JAALAG000000000
M-19	JAALFB000000000	M-3-X3	JAALBB000000000	M-S69-X3	JAALAF000000000
M-20	JAALFA000000000	M-4-X3	JAALBA000000000	M-S87-X3	JAALAE000000000
M-21	JAALBX000000000	M-7-X3	JAALAZ000000000	M-DH5a-X3	JAALAD000000000
M-22	JAALBW000000000	M-10-X3	JAALAY000000000	3R_Chromosome	CP049348
M-23	JAALBV000000000	M-11-X3	JAALAX000000000	3R_Plasmid1	CP049349
M-26	JAALBU000000000	M-13-X3	JAALAW000000000	3R_Plasmid2	CP049350
M-27	JAALBT000000000	M-14-X3	JAALAV000000000	3R_Plasmid3	CP049351
				3R_Plasmid4	CP049352

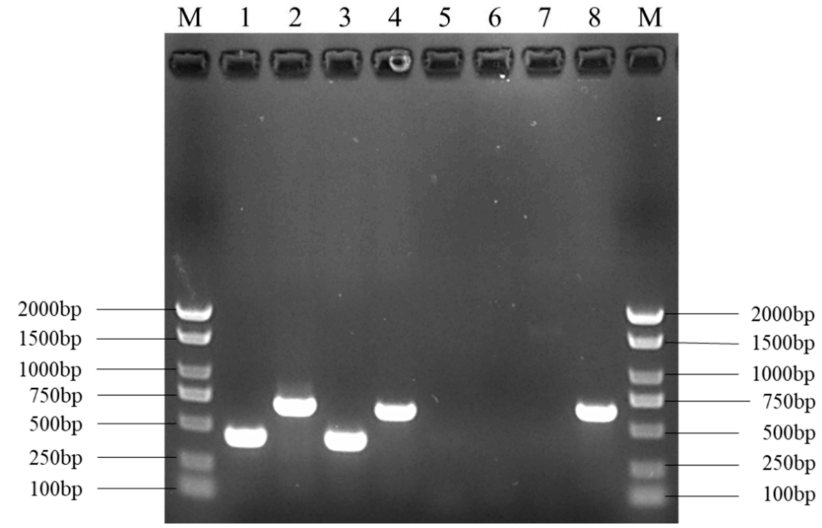

Figure 1. Verification of the IncX3 plasmid. Agarose gel electrophoresis of products amplified from the four plasmids found in *Escherichia coli* strains 3R and IncX3+DH5α. Lane M, Biomed BM2000+; lanes 1–4, DNA from strain 3R was used as template; lanes 5–8, DNA from strain IncX3+DH5α was used as template; lanes 1 and 5, primer pair TEM-1 (P1-1)-F/R was used to detect Plasmid 1; lanes 2 and 6, primer pair CMY-2 (P2-1)-F/R was used to detect Plasmid 2; lanes 3 and 7, primer pair Rep (P3-1)-F/R was used to detect Plasmid 3; lanes 4 and 8, primer pair NDM (P4-1)-F/R was used to detect Plasmid 4.

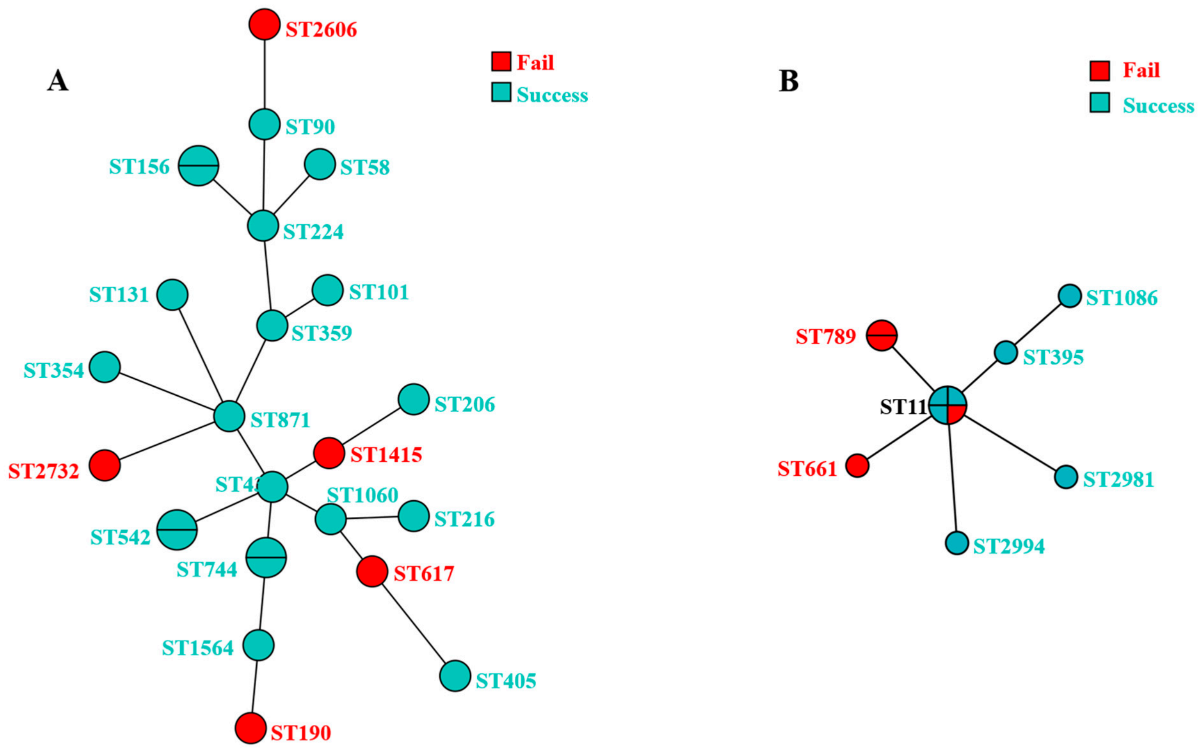

Figure S2. MLST-based clustering of recipient strains. (**A**) MLST cluster of the *Escherichia coli* recipient strains. (**B**) MLST cluster of the *Klebsiella pneumoniae* recipient strains. Green indicates successful conjugative transfer, while red indicates unsuccessful conjugative transfer.

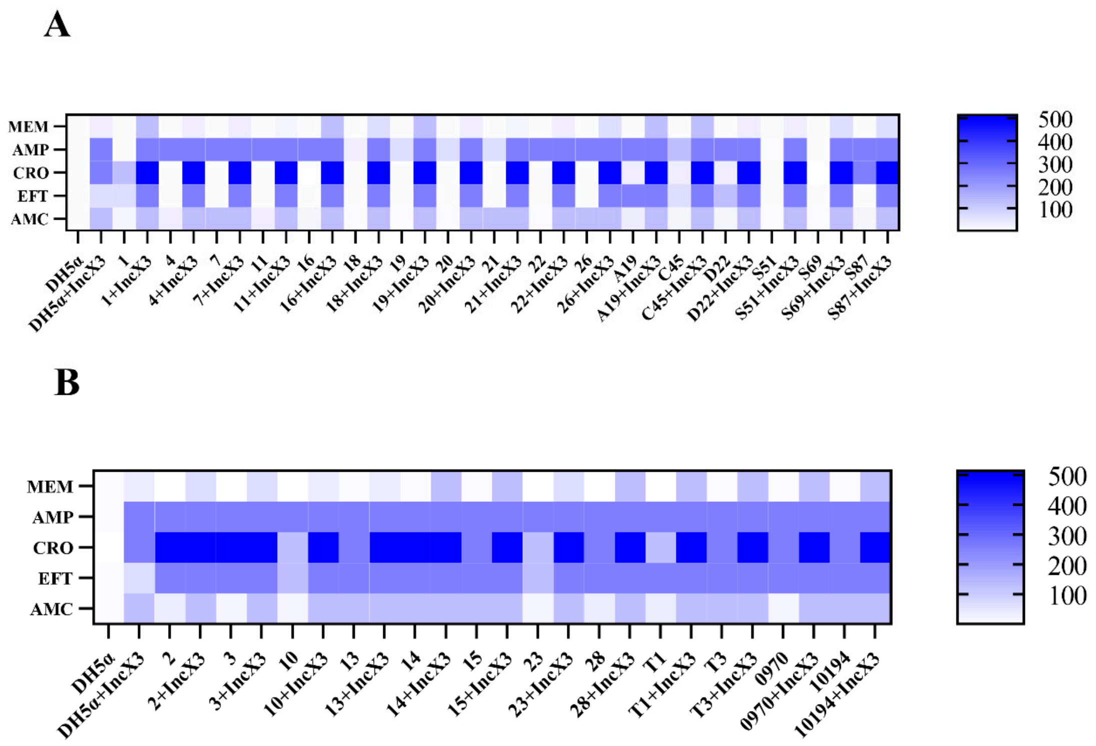

Figure S3. Changes in antimicrobial susceptibility profiles as a result of IncX3 plasmid acquisition. The heatmap indicates antimicrobial susceptibility changes among recipients after obtaining the IncX3 plasmid. Recipients shown in (**A**) were susceptible to the five β-lactam antibiotics, while the recipients in (**B**) were resistant. The darker the blue, the higher the MIC. MEM, meropenem; AMP, ampicillin; CRO, ceftriaxone; EFT, ceftiofur; AMC, amoxycillin/clavulanic acid.

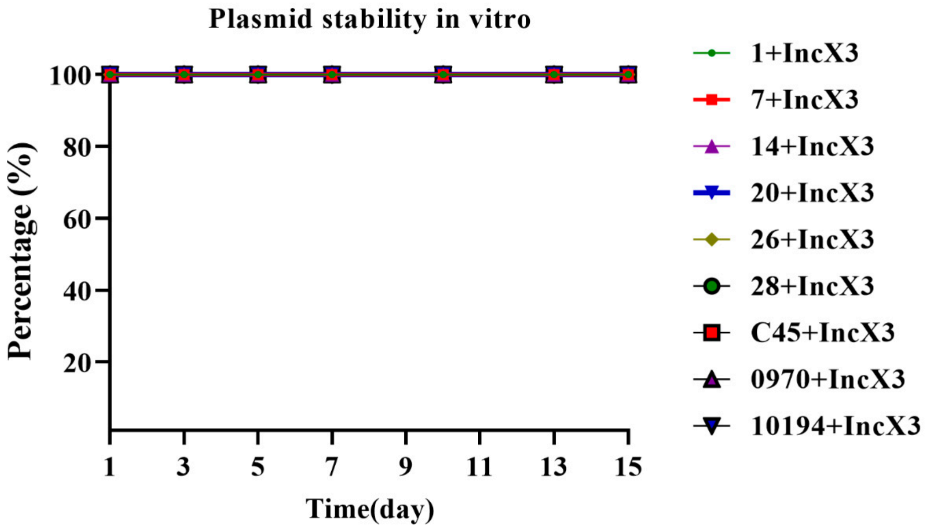

Figure S4. Stability of the IncX3 plasmid. Transconjugants from different sources were passaged continuously for 15 days in the absence of antibiotic pressure. Strain No. 1, ST354 *Escherichia coli* isolated from chicken manure compost; strain No. 7, *Enterobacter cloacae* isolated from chicken manure compost; strain No. 14, ST395 *Klebsiella pneumoniae* isolated from a chicken cloaca swab; strain No. 20, *K. pneumoniae* isolated from pork; strain No. 26, ST724 *Enterobacter hormaechei* isolated from a fly at a swine farm; strain No. 28, ST131 *E. coli* isolated from a human; strain C45, ST216 *E. coli* isolated from swine; strain 0970, ST405 *E. coli* isolated from a companion animal.
